# Comparison of self-collected versus clinician collected cervicovaginal specimens for detection of high risk human papillomavirus among HIV infected women in Ethiopia

**DOI:** 10.1186/s12905-022-01944-2

**Published:** 2022-09-01

**Authors:** Agajie Likie Bogale, Tilahun Teklehaymanot, Jemal Haidar Ali, Getnet Mitike Kassie, Girmay Medhin, Ajanaw Yizengaw Baye, Amelework Yilma Shiferaw

**Affiliations:** 1 Program of Tropical and Infectious Diseases, Aklilu Lemma Institute of Pathobiology, Addis Ababa University, Addis Ababa, Ethiopia; 2 School of Public Health, Addis Ababa University, Addis Ababa, Ethiopia; 3 International Institute of Primary Health Care, Addis Ababa, Ethiopia; 4 Ethiopian Public Health Institute, Addis Ababa, Ethiopia

**Keywords:** Cervical cancer, Cervicovaginal sampling, HPV DNA test, Ethiopia

## Abstract

**Background:**

In order to meet the WHO 2030 cervical cancer elimination program, evaluation and utilization of sensitive testing method, and feasible sampling technique is a paradigm for enhancing cervical cancer screening coverage. Self-sampling for screening of HPV DNA testing is one of the easiest and sensitive techniques, though the evidence was limited in the Ethiopian context. This study aimed to compare the performance of self-collected vaginal specimen versus clinician collected cervical specimen for detection of HPV among HIV positive women in Ethiopia.

**Methods:**

We conducted a comparative cross-sectional study design to collect cervicovaginal specimens among HIV positive women of age older than 24 years. Data were collected from six government hospitals from January to October 2021. A total of 994 cervicovaginal specimens was collected by clinicians and HIV positive women themselves in the cervical cancer screening unit using Abbott Cervi-Collect Specimen Collection Kit, and molecular HPV testing was conducted. Data were entered into an Excel spreadsheet and analyzed using SPSS version 25. Sensitivity, specificity and kappa were reported with *p* < 0.05 considered as statistically significant.

**Results:**

The prevalence of high-risk HPV was 29.4% among self-sampled specimen and 23.9% among clinician collected specimens. The overall concordance of the test result was 87.3%. Oncogenic HPV types, other than HPV16&18 were predominant in both sampling techniques, 19.9% from vaginal self-collected specimen and 16.7% of clinician collected cervical specimens. The sensitivity and specificity of self-sampled HPV test was 84.0% and 88.4%, respectively. The level of agreement was good (k = 0.68) and statistically significant (*p* < 0.001). The discriminatory power of the test as true positive and negative was excellent with an area under the curve of 0.86.

**Conclusion:**

The magnitude of oncogenic HPV was higher in self-collected samples than the clinician collected specimen with good agreement between the two sampling methods. Thus, we recommend the Ministry of Health in Ethiopia to expand utilization of the self-sampled technique and enhance the coverage of screening in the country.

## Background

Since the 1970s, several countries instituted screening and treating cervical cancer programs, however, still significant gaps and challenges persist in reducing morbidities and mortalities from the disease [1]. As evidenced in the recent report of the Global Cancer Observatory (GLOBOCAN, 2020), each year the occurrence of new cases of cancer of the cervix uteri was 604,127 with 341,831 deaths worldwide [2], and reported as the leading cause of cancer deaths in developing countries [3]. In Ethiopia, the number of new cases of cervical cancer were 7445 and 5338 deaths per annum [2].

Since the burden of cervical cancer is increasing, how does the global strategy to eliminate cervical cancer by 2030 is progressing so far? The pillar of the elimination program is set-forth 90–70–90 programmatic directions, meaning 90% of girls fully vaccinated with the human papillomavirus (HPV) vaccine by age 15 years, 70% of women are screened with a high-performance test, and 90% of women identified with the cervical disease receive treatment [4].

Per the strategy of screening using high-performance test and easy technique, what did previous reports about self-sampling for HPV detection tell us? According to a study, carcinogenic HPV infections were identified in 23.2% of patient collected specimens as compared to 34.9% of the samples collected by the clinician, with more important carcinogenic HPV types being identified by the clinician collected samples [5], and most women felt that the samples collected by clinicians were more reliable than self-collected, although, those women had little knowledge of cervical cancer and the level of education was low [6]. In contrast, the better prevalence of HPV-Deoxyribonucleic acid (DNA) was reported in self-collected specimen (22.8%), as compared with clinician-collected samples (19.2%) with a fair agreement (kappa value (k) = 0.34) [7]. In addition, self-collected samples are as good as clinician-collected samples for HPV detection and inexpensive methods [8]. In another study, there was no significant difference between professionally collected and self-collected samples for HPV detection [9]. A review study even showed that high levels of acceptance of self-sampling were observed due to the privacy and simplicity of the method [10] and had a positive attitude [11]. A study reported HPV testing using self-sampling as a novel approach to enhance screening rates, mainly in low-and middle-income countries [12].

The aforementioned results, however, had reported some controversies and necessitate further study. In addition, such kind of studies was very limited in Ethiopia. And, therefore, the current study was aimed to examine the performance of self-collected vaginal specimens versus clinician collected cervical specimens, and to set forth a guideline, and recommend a very sensitive method with an easy sampling technique in the country which is supposed to enhance coverage screening.

## Methods

### Study design

We conducted a comparative cross-sectional study to collect cervicovaginal specimens among HIV positive women of age older than 24 years in Addis Ababa, Ethiopia.

### Study setting and period

This study was conducted among selected government hospitals that employed the new HPV DNA molecular method for screening cervical cancer from January to October 2021. The facilities enrolled included the Zewditu Memorial Hospital, St. Paul Hospital, ALERT hospital, Minilik Hospital, Yekatit-12 and St. Peter Hospital. The Zewditu Memorial Hospital is located in the Kirkos sub-city, St. Paul’s Hospital and St. Peter Hospital are located in the Gulele sub-city, Yekatit-12 and Menelik-II Hospitals in Arada sub-city, Alert Hospital located in Kolfe Keranio. Ethiopia is a country in the horn of Africa, and has eleven Regional States (Tigray, Afar, Amhara, Oromiya, Southern Nations, Nationalities and peoples’ region (SNNPR), BenishangulGumuz, Gambela, Sidama, Somalia, Harari, and South West) and two City administrations (Addis Ababa and Dire Dawa). Addis Ababa is the capital city of the country and it is one of the two city administrations. The city is divided into 11 sub-cities, and geographically located at 9°1′48″N38°44′24″E, covering an approximate land area of 526.46 square km and lying at an elevation of 2355 m (7726 feet) [13].

### Inclusion and exclusion criteria

All human immunodeficiency virus (HIV) positive women that had antiretroviral therapy (ART) follow-up and age older than 24 years, who volunteered to participate in the study, and signed consent were counseled and included in the study. However, pregnant women, women in menstrual period during the time of specimen collection, no history of sexual activity, received pelvic radiation therapy, period of genital tract infection (e.g., vaginitis) or acute inflammation, poor labeling or mislabeling of the specimen were the exclusion criteria used.

Considering all the eligibility requirements, a total of 497 self-collected specimens and about the same number of clinicians collected cervical specimens with an overall total of 994 specimens collected from both groups for molecular HPV testing.

### Data collection and test procedure

Data were collected by trained clinicians and participants themselves for self-sampling. Intensive counseling was given to HIV positive women attending cervical cancer screening unit on self-sampling. Two transport tubes were used to collect cervicovaginal specimens for each woman (one for self-sampling and another for clinician’s collection) and were labeled. The tube labeled self (s) and one cervical brush were given to the woman to collect the specimen. Then clinicians collected cervical specimen using speculum examination immediately. After cervicovaginal sample collection, screening using visual inspection with acetic acid (VIA) was done by trained staff. The specimens were collected using Abbott Cervi-Collect specimen collection kit (List No. 4N73). Each kit contains; One Cervical Brush and one Transport Tube containing 2.4 ml Specimen Transport Buffer (guanidine thiocyanate in a Tris buffer) which stabilize DNA till sample preparation. The collected specimens were transported to the testing laboratory, and trained laboratory professionals and the principal investigator conducted laboratory testing using Abbott real time polymerase chain reaction (PCR) (m2000rt/SP) instrument and its molecular HPV testing assays (ABBOTT Max-Planck-Ring 2, 65205 Wiesbaden, Germany). The principles of the procedure were described in a previous publication [14].

### Quality assurance

Training was given for data collectors and the quality of data was maintained with continuous follow-up on the process. The recruited data collectors were experienced in conducting cervical cancer screening and treatment. The laboratory test was done by trained lab professionals and the principal investigator who are working at the accredited laboratory by the Ethiopian National Accreditation Office (Accreditation number, M0030). The positive and negative external quality control included in each run to monitor the quality of test parameter. The entire process from extraction to amplification and detection was maintained using the endogenous human beta globin, internal control (IC).

### Data entry, processing and analysis

The collected data were entered into an Excel spreadsheet, coded and exported to SPSS version 25 for analysis. Descriptive statistics were applied to determine the performance measures, including sensitivity, specificity and kappa values. According to Altman (1991), the interpretation of the kappa value or the strength of the agreement was reported as poor (< 0.2), fair (0.2–0.4), moderate (0.4–0.6), good (0.6–0.8), and very good (0.8–1.0) [15]. The sensitivity and specificity of HPV self-sampling were calculated using clinician collected samples as the standard [16], with the assumption that specimen collection by trained professionals had higher accuracy than self-collection, despite the fact that self-collection was done after relevant counseling of participants on how to collect self-sample. We also applied receiver operating characteristic curve (ROC) and area under the curve (AUC) to see the performance measures of a comprehensive evaluation of classification models, as true positive and false positive rates, and its discriminatory power. An AUC of 0.5 proposes no discrimination, 0.7–0.8 is acceptable, 0.8–0.9 is excellent, and over 0.9 is outstanding [17]. For all analysis *p* < 0.05 was considered statistically significant.

## Results

Of the total 497 women recruited in this study, 994 specimens were collected, both vaginal and cervical. From the given total, 16 (3.2%) samples were missing. Of which two specimens were failed lab result and the remaining’s were with no HPV DNA result recorded on the worksheet due to mislabeling from sample transaction sheet and sample tube. Demographic characteristics of the participants were described in a previous publication elsewhere [14].

The findings of clinician collected specimen indicated that 363(73.0%) HIV positive women were negative for HPV and 119 (23.9%) were high-risk (HR) HPV positive, whereas, the finding of self-collected vaginal specimens indicated 349 (70.2%) HIV positive women were negative for HPV while 146 (29.4%) were HR HPV positive women. More frequent oncogenic HPV types identified in both specimen types were non-HPV16 and 18 (other HR HPV), i.e., 16.7% (83/497) from clinician collected cervical specimens vs. 19.9% (99/497) from vaginal self-collected specimen. In addition, 3.8% (19/497) oncogenic HPV16 from clinician collected vs. 4.8% (24/497) of self-sampled, and mixed HPV16 and other HR HPV of 2.4% (12/497) vs. 3.2% (16/497) of clinician and self-collected specimens, respectively of HPV burden were observed. One self-collected specimen (0.2%) indicated oncogenic HPV16, 18 and other HR HPV types (Table 1).

**Table 1:** The distribution of high-risk HPV in both self-collected and clinician collected specimens in Ethiopia

HPV type	Specimen type	
	Cervical specimen N (%)	Vaginal specimen N (%)
HPV16	19 (3.8%)	24 (4.8%)
HPV16 and 18	1 (0.2%)	1 (0.2%)
HPV16 and other HR HPV types	12 (2.4%)	16 (3.2%)
HPV18	3 (0.6%)	3 (0.6%)
HPV18 and other HR HPV types	1(0.2%)	2 (0.4%)
other HR HPV(non-HPV16 and 18)	83 (16.7%)	99 (19.9%)
HPV16, 18 and other HR HPV types	0	1 (0.2%)
Total	119 (23.9%)	146 (29.4%)

N = number, and percent (%)

In general, other HR HPV types were highly detected in this cervicovaginal specimen that need further investigation. The overall concordance result was identified in 87.3% (420/481) of the cervicovaginal specimens tested. In addition, 12.7% (61/481) discordant results were identified which need further investigation.

The sensitivity and specificity of HPV self-sampled testing were 84.0% and 88.4%, respectively. The level of agreement was statistically significant (*p* < 0.001) and a good agreement was observed in both sampling methods with k = 0.68 (Table 2).

**Table 2:** The sensitivity and specificity of cervicovaginal specimen for the molecular detection of high-risk HPV in Ethiopia

	Clinician collected specimen			Measure of agreement	
	Negative	Positive	Total	Kappa value (k)	*p* Value
*Self-collected specimen*					
Negative	320(88.4%)	19(16.0%)	339(70.5%)	0.68	0.001
Positive	42(11.6%)	100(84.0%)	142(29.5%)		
Total	362(100%)	119(100%)	481(100%)		

**Fig. 1:**
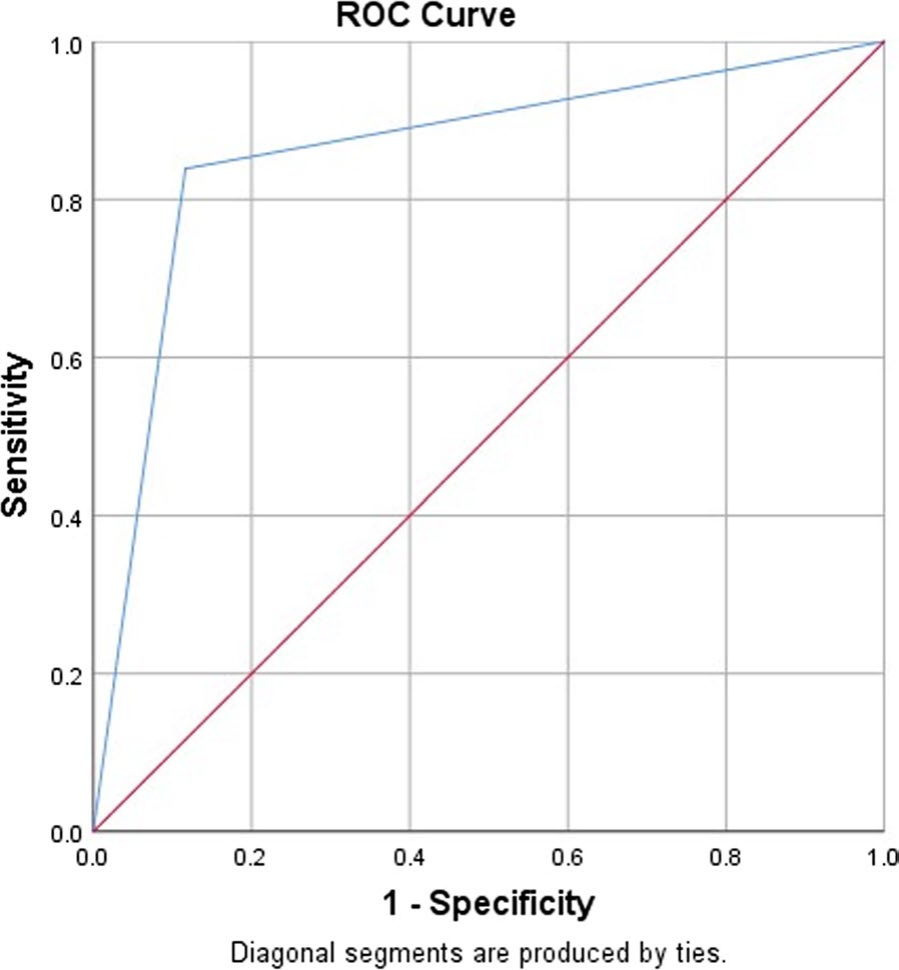
The true positive rate and the false positive rate as a measure of performance for provider collected versus self-collected specimen for high risk HPV detection. The sensitivity in the y-axis indicates the true positive rate and 1-specificity in x-axis indicates false positive rate

In this study, we tried to see the performance measures of a comprehensive evaluation of classification models, considering ROC and AUC. The true positive rate called sensitivity on the y-axis and the false positive rate (1-specificity) on the x-axis were also measured (Fig. 1). Thus, sensitivity was 0.84 and 1-specificity was 0.116 of the cutoff point 0.5, meaning the true positive and the false positive rates of the method were 84.0% and 11.6%, respectively. The AUC was 0.86 with the 95% CI (0.82–0.91; *p* < 0.001), indicating that the discrimination power of the test method as true positive and negative results was excellent.

## Discussion

The HPV testing using self-sampled specimen was reported as highly accepted, comfortable, and safe [18, 19], though, the accuracy of new combinations of assays and self-sampling devices has been evaluated in a diagnostic setting [20], and clinical performance should be identified [21]. In this study, we estimated the detection of HR HPV from clinician collected cervical specimens and self-collected vaginal specimen among HIV positive Ethiopian women, and the key findings were as follows: The magnitude of HR HPV using self-sampled vaginal specimen was 29.4%, which was higher than clinician collected cervical specimen, indicated 23.9%. The sensitivity and specificity HPV test using self-sampled specimen as compared to the clinician collected specimen was 84.0% and 88.4%, respectively. A good agreement was observed between the two sampling techniques with k = 0.68.

A study in India indicated that the prevalence of HPV DNA was high, 78.1% of cervical samples and 77.2% in vaginal samples, and the overall agreement between the two sampling methods was 93.9%, and [22]. The high positivity rate of clinician collected and self-collected samples were reported in Japanese patients, which was 51% and 50%, respectively, with k = 0.76 [16]. In addition, more significant oncogenic HPV types were identified by other authors from clinician collected specimen as compared to patient collected specimens with a fair agreement (k = 0.45) [5]. A Ghanaian study depicted that the overall concordance of HPV detection of self-and clinician collected specimen was 94.2% showing excellent agreement (k = 0.88) [23]. The aforementioned findings were higher than our current finding where the prevalence of oncogenic HPV infection in the self-sampled specimen was (29.4%) as compared with clinician collected (23.9%). On the contrary, in the Netherlands a study depicted that the prevalence of HR HPV was lower in both sampling methods which was 8.0% among clinician collected samples and 10.0% among self-samples, although the concordance of HR HPV prevalence between self-collected and clinician collected samples was high, 96.8% [24]. A study conducted in Nigeria indicated that, there was moderate agreement between self-and clinician collected samples in the detection of HPV (k = 0.47) with the HR HPV detection rate of 7.2% in self-sampling and 6.8% in clinician collected specimens [25]. In Tanzania it was reported that the prevalence of HR HPV was lower in self-collected sample compared with clinician collected specimen (13.8% vs. 19.0%), though there was a good overall agreement 90.5% [26]. In Ethiopia, an overall HR HPV prevalence of 17.2% self-collected specimens and 15.5% of the clinician collected specimens with a moderate agreement (k = 0.58) was reported [27]. Another study also indicated that the prevalence of HR HPV from self-collected specimens was 14.0% [28].

The overall differences observed in the findings of the studies were the difference in the number and characteristics of the study participants, and the instrument used for molecular HPV testing. For instance, the gene for amplification was based on the long control region (LCR) and early proteins, E6/E7 and the PCR technique was different [22], and the participants involved in the study were cervical intraepithelial neoplasia2 and worse (CIN2+) patients [16], women 18 years and older who were scheduled for colposcopy examination following an abnormal screening or surveillance of post therapeutic conization, and self-collection made without the assistance of medical personnel [5], and also the number of participants and participants characteristics [23], which was different from our study requirements. Meaning, analytical sensitivity and/or target gene amplified in different molecular techniques, the persistence of HPV infection in older age, and other factors discussed in the previous paper [14], might contribute to the difference in the reported findings mentioned above. In addition, the quality assurance aspect in the molecular genetics laboratory might induce the observed differences [29, 30]. The sample collection experience, mainly self-sampling might vary in different countries and/or population [31]. And also, risk factors for HPV infection like poor and/or inadequate personal hygiene and care, lack of gynecological examination history, education level, unprotected sex, and others might account for the variations in settings and populations [32].

A review paper in low- and middle-income countries revealed that the concordance rate of self-sampling with clinician collected sampling was 87.0–97.5% [12], which was closer to our current finding that indicated 87.3% concordance.

The Indian study depicted that the sensitivity and specificity of both self-sampling and clinician collected specimen were more than 98.0% [22]. Similarly, the Ghanaian study indicated that the sensitivity and specificity in both sampling techniques were more than 90.0% [23], while the study in Tanzania reported sensitivity and specificity of 61.4% vs. 97.3%, respectively [26]. The current finding with a sensitivity of 84.0% and specificity of 88.4% was closer to the previous study findings. The sensitivity of self-sampling was comparable to liquid-based cytology technique and superior to VIA [33].

Oncogenic HPV types were detected with higher prevalence of self-samples than clinician collected cervical specimen in the current study, of which, HR HPV types other than HPV16 and 18 were predominant, 19.9% of self-collected specimens versus 16.7% of clinician collected specimens. The reported finding on the genotypic distribution of HR HPV types other than HPV16 and 18 were similar with the previous finding in Ethiopia [34]. Another study among Ethiopian women depicted that the commonest HPV genotypes were HPV16 followed by HPV52 and HPV58 [35]. This highlights further genotyping of available specimens to investigate the commonest oncogenic HPV types of the predominant non-HPV16 and 18 in our study.

The women preferred self-sampling compared with clinician collected samples primarily due to privacy, although nurse assistance was needed [36]. When compared with pap test, self-sampling is more acceptable [37], and easy to do [38] with no difficulty [39]. Studies indicated that HPV self-sampling was the potential to increase the coverage of cervical cancer screening [40] and highly accepted cervical cancer screening method for end users [41, 42], that complements recent screening program by increasing population coverage [33]. In addition to the comparability of self-sampling with clinician collected specimens, self-sampling was feasible and accurate means of obtaining samples from low resource settings [20, 43]. Besides identifying high risk and low risk HPV types, self-collected specimens were equally important for microbiome analysis [31].

Implementing sensitive screening modalities combined with easy sampling methods with minimum pain or discomfort, such as self-sampling of vaginal and urine samples are highly encouraged and widely supported by the scientific community [44]. Our finding might complement the recommendations and is a widely supported sampling method (self-sampling) for the screening of cervical pre-cancer and cervical cancer in general. This sampling method might resolve the fear of pain, shyness of women and resources like the use of a speculum for cervical specimen, cleaning and sterilization procedure of materials. The method was good, even to screen postmenopausal women with a difficulty to take cervical specimen. Surprisingly, one study participant had a sexual contact within eleven hours during the time of data collection, and both clinician collected cervical and vaginal self-collected specimens were taken. The molecular test result indicated that cervical specimen collected by clinicians had positive for other HR HPV type and self-collected vaginal specimen had negative for HR HPV. This finding highlighted that, self-collected vaginal specimen might not be appropriate for HPV testing of the specimen collected within 24 h of sexual contact, while cervical specimens might be appropriate in such scenario not to appoint women for other days to collect the specimen. Considering overall eligibility requirements and awareness creation strategies, self-sampling for HPV detection in general, might enhance the coverage of cervical cancer screening in Ethiopia.

### Strength and limitation of the study

The HR HPV detection using a real time PCR among vulnerable population, which was women with HIV with sufficient sample size in a resource limited setting, and the highlighting of the importance of self-sampling in the population of Ethiopia might be taken as a strength of the study. However, the study had limitations, that include, lack of further clinical investigation and follow-up of HPV positive women to clearly identify the probability of oncogenic HPV that causes cervical cancer.

## Conclusion

The magnitude of oncogenic HPV was higher from self-collected samples than the clinician collected specimen with good agreement between the two sampling methods. Therefore, the findings of the study may be considered by the Ministry of Health in Ethiopia and its stakeholders to expand the utilization of the self-sampling method in the country. Self-sampling might increase the screening coverage at national level to meet the World Health Organization (WHO) 2030 global cervical cancer elimination program.

## Data Availability

All data generated or analysed during this study were included in this published article.

## Abbreviations

ART: Antiretroviral therapy
AUC: Area under the curve
CIN: Cervical intraepithelial neoplasia
DNA: Deoxyribonucleic acid
HIV: Human immunodeficiency virus
HPV: Human papilloma virus
HR: High risk
IC: Internal control
k: Kappa value
LCR: Long control region
Pap: Papanicolaou
PCR: Polymerase chain reaction
ROC: Receiver operating characteristic curve
VIA: Visual inspection with acetic acid
WHO: World Health Organization
